# Cerebral Palsy: A Narrative Review on Childhood Disorder

**DOI:** 10.7759/cureus.49050

**Published:** 2023-11-19

**Authors:** Sakshi Basoya, Sunil Kumar, Anil Wanjari

**Affiliations:** 1 Medicine, Jawaharlal Nehru Medical College, Datta Meghe Institute of Higher Education and Research, Wardha, IND

**Keywords:** dyskinesia, muscle spasticity, types of cerebral palsy, treatment of cerebral palsy, cerebral palsy (cp)

## Abstract

Cerebral palsy, one of the most common reasons for infirmity in children and young people in developed countries, refers to several neurological diseases that impact movement and coordination. Central nervous system damage received during the first stages of brain development can cause cerebral palsy, a non-progressive condition that manifests as impairments of movement and posture. Two cases per 1000 are reported, and the causes include those mentioned for high-risk infants. Mental retardation, sensory deficiencies, failure to thrive, seizures, and behavioral or emotional issues are some of the associated difficulties. To enable interdisciplinary intervention, early identification is crucial. The result varies depending on the topography, severity, and presence of concomitant abnormalities in cerebral palsy. Cerebral palsy is caused by a static injury to the cerebral motor cortex that happens before, during, or within five years after birth. Various circumstances can influence the disease, including cerebral anoxia, cerebral hemorrhage, infection, and hereditary disorders. Interventions for children are typically provided as part of multidisciplinary rehabilitation programs. Musculoskeletal complaints are common, and pain is a significant underreported symptom.

## Introduction and background

Cerebral palsy, a leading factor in childhood impairment, is one of a variety of non-progressive postural and motor dysfunction syndromes [1,2]. Recent definitions allow physicians to understand more than simply the movement problem that arises from an irreversible injury to the developing brain [3,4]. Historically, cerebral palsy has been defined as a condition of movement and posture [5]. Complementary and alternative therapies are frequently used by families treating their progeny with cerebral palsy holistically; nevertheless, the prevalence of their usage and the price of these alternatives remain unknown [6,7]. Cerebral palsy is predominantly a mobility issue, but many children who have it also have additional disabilities that may lower their quality of life and shorten their lifespan [8]. Spasticity is one of the defining problems of cerebral palsy [5]. Reduced movement control, weakness, lassitude, atypical tone, atypical posture, bone deformity due to the development of muscle contracture, and pain of increased intensity that may occur during active or passive movement as well as a result of flexor or extensor spasms are some of the characteristics of upper motor neuron syndrome [5]. The four subtypes of cerebral palsy-related movement disorders include ataxia, mixed/other, dyskinesia, and spasticity. The most prevalent mobility difficulty in 80% of kids with cerebral palsy is spasticity [9,10]. The aim of the article is to spread knowledge about cerebral palsy and available treatment modalities in society.

## Review

Search methodology

**Figure 1:**
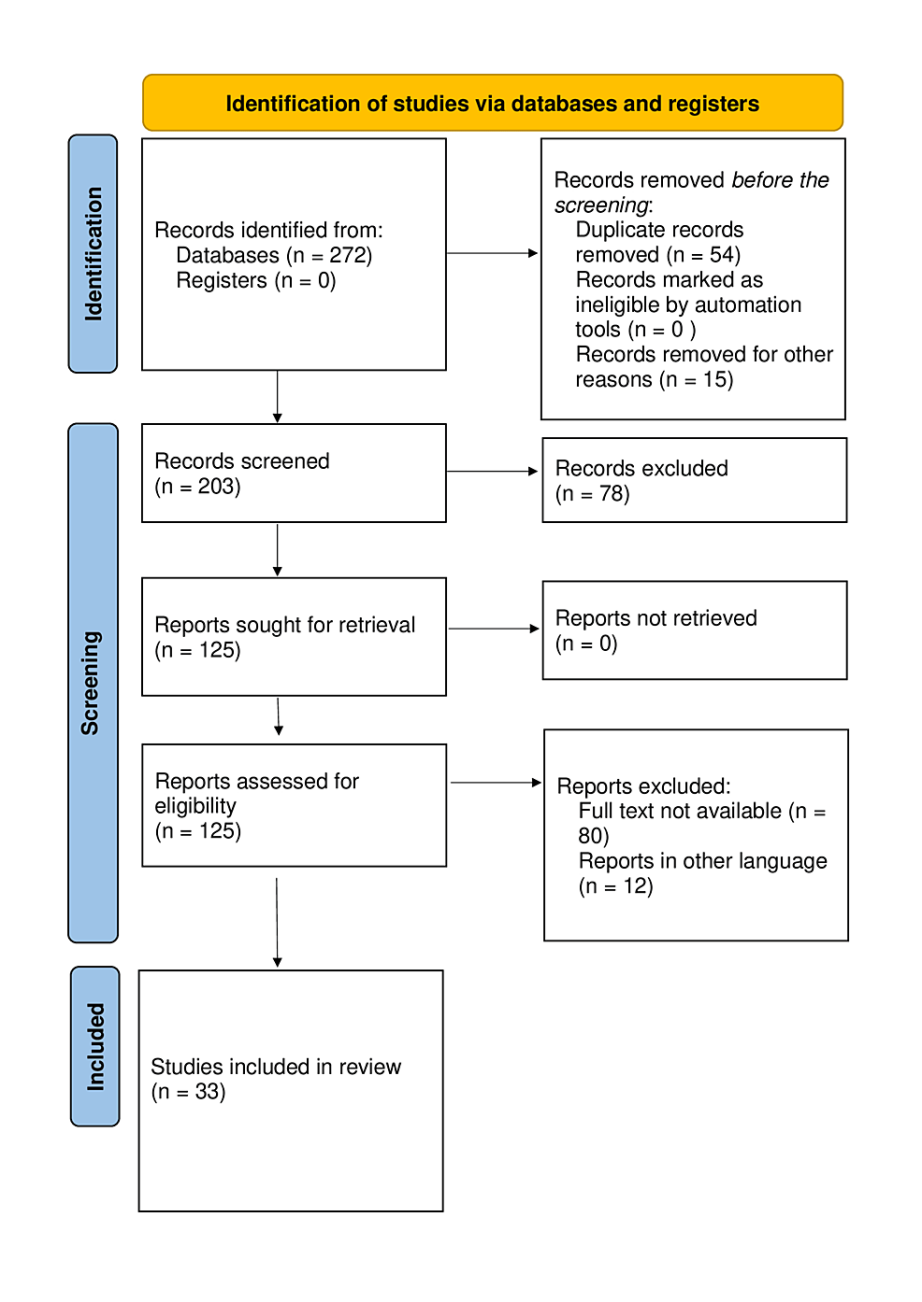
Prismatic flow diagram of cerebral palsy Adopted from the preferred reporting items for systematic reviews and meta-analysis (PRISMA)

We undertook a systematic search through PubMed, Google Scholar, National Library of Medicine, Proceedings of the National Academy of Sciences and Central in May 2023 using keywords such as “Cerebral palsy”, “musculoskeletal complaints”, “spastic tetraplegia”, “spasticity”, and “sensory deficits” (((Cerebral palsy (Title/Abstract)) OR (Cerebral palsy (MeSH Terms))), (musculoskeletal complaints (Title/Abstract)) OR ((musculoskeletal complaints (MeSH Terms)), (spastic tetraplegia (Title/Abstract)) OR (spastic tetraplegia (MeSH Terms)) and (spasticity (Title/Abstract)) and (spasticity (MeSH Terms)). The selection of the studies depended on the following inclusion criteria: (1) cerebral palsy (its occurrence in children); (2) pattern of presentation in children; (3) musculoskeletal complaints; and (4) English language. The following were the exclusion criteria: (1) case study; (2) animal studies; (3) bench research; (4) not an empirical study (e.g., theory or opinion articles); and (5) non-English language research. Figure 1 shows preferred reporting items for systematic reviews and meta-analysis flow diagram for a literature search.

Etiology of cerebral palsy

**Figure 2:**
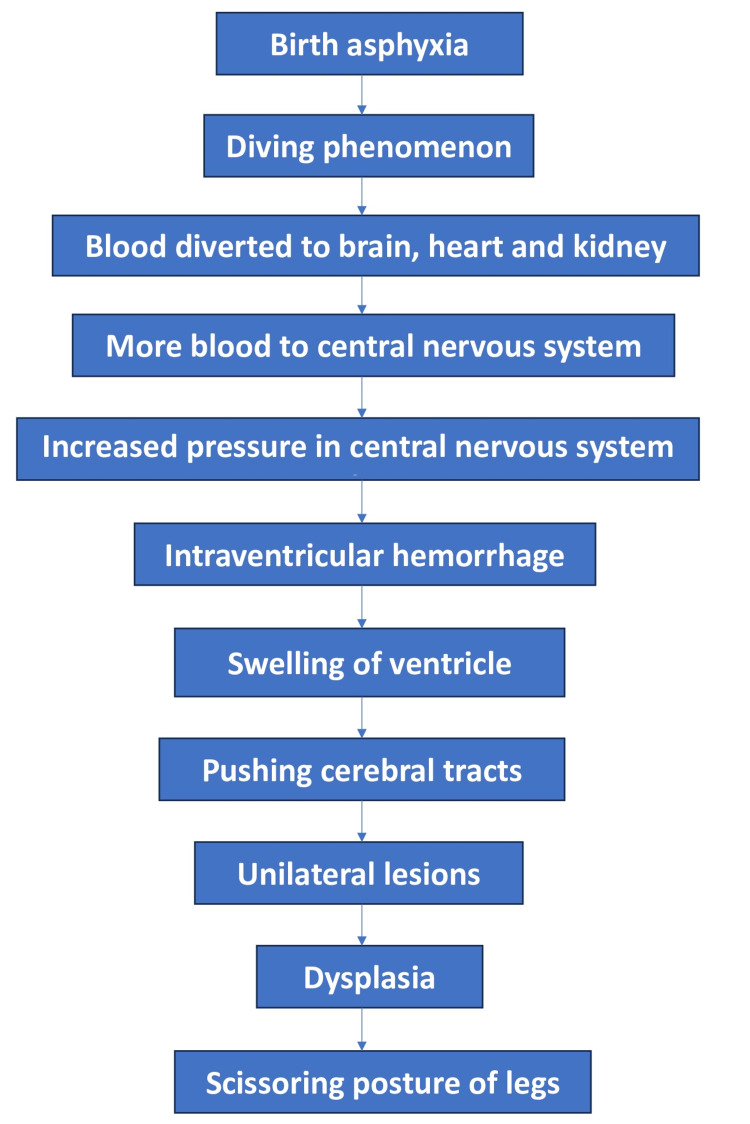
Pathophysiology of Cerebral palsy Credit: Image created by the author

Brain damage or improper prenatal or neonatal brain development causes cerebral palsy. The non-progressive ("static") condition known as cerebral palsy can appear before, during, or after childbirth. The etiology of a patient is usually complex [11]. These causes can be separated into three categories: prenatal, perinatal, and postnatal causes. Some of the prenatal diseases known to play a part in the development of cerebral palsy include chromosomal abnormalities, intrauterine infections, intrauterine stroke, and congenital brain malformation [12]. The perinatal causes of cerebral palsy include hypoxic-ischemic insults, central nervous system (CNS) infections, strokes, and kernicterus [13]. In the category of postnatal causes, there are both accidental and unintentional CNS infections, strokes, and anoxic insults [11]. Cerebral palsy is far more likely to occur in premature babies. Additional risk factors for cerebral palsy in multiple pregnancies include intrauterine growth restriction, maternal drug abuse, hypertension, chorioamnionitis, aberrant placental pathology, meconium aspiration, newborn hypoglycemia, and genetic predisposition [14,15]. Figure 2 shows the pathophysiology of cerebral palsy in a flow diagram manner.

Classification of cerebral palsy

According to the kind of movement issue that is present (spastic, athetoid, ataxic, or mixed), the body parts affected (hemiplegia, diplegia, or quadriplegia), or the functions impacted (mild, moderate, severe, or profound), cerebral palsy is frequently categorized [4]. Spastic cerebral palsy comes in many types depending on the body parts affected [16]. In spastic hemiplegia or hemiparesis unilateral side of the body is affected by the arm, hand, and occasionally the leg. While intellect is often normal, children of this kind may experience delays in their ability to speak. Spastic diplegia or diparesis causes less damage to the arms and face in people of this kind, who often suffer muscular stiffness in the legs [17]. Language proficiency and intelligence are typically average. Spastic quadriplegia or quadriparesis is the most severe kind of cerebral palsy, characterized by a floppy, or weak, neck and extreme rigidity in the limbs and legs. Spastic quadriplegics are typically unable to walk and frequently have speech difficulties. Intellectual or developmental disability of this kind can range from mild to severe.

Dyskinetic cerebral palsy is a kind that entails erratic hand movements, feet, arms, or legs that are sluggish and out of control [16]. Some kids may drool or make faces due to hyperactive facial and tongue muscles. People with this kind frequently struggle to walk or sit upright. Intellectual difficulties are typically absent in people with dyskinetic cerebral palsy. Ataxic cerebral palsy has an impact on balance and depth perception. When walking or performing rapid or precise actions like writing, buttoning a shirt, or reaching for a book, people with ataxic cerebral palsy have difficulty [16]. In mixed types of cerebral palsy, the symptoms overlap with those of the other types which are spastic, dyskinesia, and ataxic.

Clinical presentation of cerebral palsy

**Figure 3:**
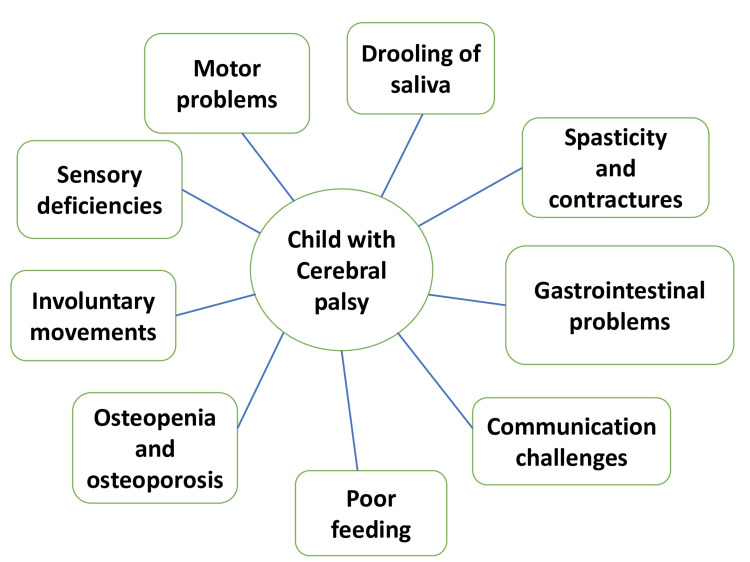
Clinical features of cerebral palsy Credit: Image created by the author Sakshi Basoya

Cerebral palsy presents with a variety of signs and symptoms, but the primary ones are motor problems, sensory deficiencies, and related comorbidities that result from static damage to the developing brain. Communication occurs between the cerebral cortex, thalamus, basal ganglia, brain stem, cerebellum, spinal cord, and communicating sensorimotor channels to normal coordination of motion [18]. This complex network is exposed to risk on many different fronts. As the kid gets older, these indications and symptoms evolve, and new traits are added to the list. These include functional gastrointestinal disorders causing bowel blockage, emesis, and difficulty in passing stool, as well as spasticity and contractures, poor feeding, salivation, communication challenges, osteopenia, osteoporosis, fractures, and pain [19]. Figure 3 shows the symptoms of cerebral palsy in children. Clinically, it is determined by a subjective physical examination, and it is frequently rated using the Modified Tardieu Scale and Modified Ashworth Scale in order to determine severity. Unwanted, regularly recurring, involuntary movements known as dyskinesias are generally caused by sickness or harm to deep brain regions.

Clinical Presentation of Hemiplegic Cerebral Palsy

Clinical features of hemiplegic cerebral palsy include decreased movement on the affected side, early hand effect (damage to the hands is more severe than that to the legs), delayed walking, hemicircumductive gait, increased muscle tone, tiptoe walking (which is caused by increased tone in the gastrocnemius muscle), Babinski is positive, and deep tendon reflexes are present.

Clinical Presentation of Diplegic Cerebral Palsy

Commando crawl is characterized by increased tone in the lower limbs, difficulty changing diapers, leg spasticity, ankle clones, and excessive hip adduction. Additionally, the Babinski sign is positive on both sides, scissoring is visible on axillary suspension, equinovarus deformity is visible, and an unproportionate increase in the size of the upper torso is present along with normal intelligence.

Clinical Presentation of Spastic Quadriplegic Cerebral Palsy

This kind of cerebral palsy is the most prevalent and severe. The characteristics of spastic cerebral palsy include upper motor neuron hypertonia, mental retardation, seizures, difficulty swallowing, increased tone in all limbs, brisk reflexes, bilaterally positive Babinski sign, flexion contraction of the ankle and wrist, delay in meeting developmental milestones, microcephaly, and speech and hearing issues that cause a difference in the child's development compared to other children of the same age.

Diagnosis of cerebral palsy

A clinical diagnosis of cerebral palsy is usually made before the age of two, with the majority of cases being identified in the first year of life [20]. Though not every symptom or indication is present in every person, it is diagnosed based on both clinical symptoms and neurological indicators. It's crucial to avoid making the diagnosis too early in infancy, especially if the symptoms are mild, as early neuromotor abnormalities might resolve, especially in preterm newborns [21]. It's critical to exclude genetic and metabolic conditions that manifest with symptoms similar to cerebral palsy. To make an early diagnosis (including eliminating masqueraders) and to help choose a course of therapy, it is critical to identify particular etiologies. The first stage in the etiologic examination is the brain MRI, which is the acknowledged standard screening evaluation for all people with cerebral palsy. About 83-86% of people with cerebral palsy have macroscopic aftereffects of brain damage or abnormalities [22]. Labeling children as having cerebral palsy only based on aberrant examination findings without supporting evidence of activity limitation is not helpful; instead, the degree of activity limitation should be described. Brain imaging can be beneficial in locating the underlying problem in the brain and occasionally offers etiologically pertinent information. A clinical assessment for cerebral palsy is typically prompted by a child's failure to meet expected developmental milestones, particularly in babies who have risk factors for the condition [23]. The existence of aberrant brain imaging is not a criterion for diagnosis, nor is it a conclusive indicator of cerebral palsy in the absence of motor symptoms [20]. In order to properly organize and carry out an early intervention program for a child with cerebral palsy, a complete neurodevelopmental assessment of the child should also evaluate any accompanying deficiencies [24].

Management of cerebral palsy

A team approach is most helpful for treating children with cerebral palsy; the team should include an orthopedist and doctor. Good sitting posture, preventing hip dislocation (spastic hip disease), and maintaining appropriate custodial care are top priorities in the non-ambulatory patient [4]. Every child is different and has differing degrees of disability. Although classification is crucial for identifying each child's disability and organizing the administration of care, each child's requirements must be considered when designing a treatment plan. Spastic cerebral palsy most frequently takes place in this type. Because of their rigid muscles, people with spastic cerebral palsy frequently make jerky motions. In order for the child with cerebral palsy to receive the greatest care possible, his or her family and community must be considered [25]. Early identification has become essential in the medical care of cerebral palsy, and it is thought that this would provide patients early access to treatments that could naturally alter the trajectory of the disorder [26]. These experts may play a number of distinct roles in looking after a patient with the disorder, depending on the setting in which they operate. Given the wide range of functional deficits and the possibility of temporal fluctuation, a customized therapeutic approach is needed. Treatment options are often symptomatic in order to promote independence, function, and/or ease of care while reducing adverse effects [25]. Only the child's epilepsy may be treated at a different hospital [27]. Adults and children should be provided primary care by physicians with the assistance of specialists in neurology, orthopedics, and rehabilitation medicine [28]. Doctors should work with them as well as educators, nurses, social workers, and rehabilitation therapists. Due to mounting evidence of neuroplasticity, the focus of rehabilitation therapy has lately switched to neurological rehabilitation. This strategy takes advantage of the brain's innate ability to evolve and adapt throughout a patient's life in order to enhance growth and function [28]. People with cerebral palsy live longer than the average population; thus, therapies must be developed to meet their needs as they age. Therefore, effective therapy often requires multidisciplinary support from families, healthcare professionals, therapists, educators, and other community members while taking cognition, language, learning, and behavior into consideration [22]. The foundation of habilitative and rehabilitative care for persons with cerebral palsy has traditionally been physical therapy and other non-pharmacologic therapies. There are many different non-pharmacologic treatments available, and more are always being created.

By blocking the sensory portion of the deep tendon reflex, the widely used neurosurgical surgery known as selective dorsal rhizotomy (SDR) reduces spasticity mostly in the legs. Children with spastic diplegia (without dystonia), which is frequently caused by periventricular leukomalacia, who are capable of participating in SDR, have strong antigravity muscles, have selective motor control, and have not responded to less invasive interventions, are most likely to benefit from extensive rehabilitation [29-31]. Electrodes are implanted during deep brain stimulation (DBS) in deep gray areas like the globus pallidus. A generator positioned subcutaneously in the upper chest regulates stimulation. More broadly, the outcome data in individuals with dyskinetic cerebral palsy are scarce and inconsistent. With some notable exceptions, DYT1 dystonia often responds well to DBS, particularly in patients with shorter disease duration and no orthopedic deformity. However, subsequent worsening of dystonia may occur [32-35]. Table 1 gives the summary of the studies included in the narrative review.

**Table 1:** Summary table for the studies included in the review DBS: Deep brain stimulation

Author name	Year of study	Summary of the publication
Dean [1]	2017	The author's study provides information on the prevalence of cerebral palsy in children in the United Kingdom.
Eicher and Batshaw [2]	1993	The research carried out by the author talks about the prevalence of cerebral palsy in high-risk infants and various associated deficits occurring due to insult to the brain.
Blair and Watson [3]	2006	The definition and multiple classifications of cerebral palsy are covered in this article, along with changes in its prevalence over time stratified by related factors and a brief summary of the most current etiological research.
Dabney et al. [4]	1997	The treatment of cerebral palsy, the benefit of computerized gait in the treatment, and the various risk factors predisposing to the condition are discussed in this article.
Kent [5]	2013	In comparison to their peers, children and young adults with physical impairments participate in less leisure activities that are more passive, domestic in nature, and lack diversity, according to a systematic study.
Koman et al. [6]	2004	The cognitive, medical, and social problems linked to cerebral palsy are only tangentially discussed in this course; just the musculoskeletal concerns are covered.
Wimalasundera and Stevenson [7]	2016	The article talks about how common cerebral palsy is, treatments that are more relevant for those who have cerebral palsy, and how their caregivers are being delivered with the support of more suitable outcome metrics that include quality of life and involvement.
O'Shea [8]	2008	The elevated likelihood of developing cerebral palsy in preterm babies as compared to full-term babies is discussed and the gross motor classification of cerebral palsy has been briefly explained.
Longo and Hankins [9]	2009	The advancement toward establishing the etiology and pathophysiology of cerebral palsy is covered in this overview.
Vitrikas et al. [10]	2020	The importance of early diagnosis and the role played by various healthcare providers are discussed in this article.
Mathewson and Lieber [12]	2015	This study explores the etiology of muscular contracture in cerebral palsy.
Rouquette and Null [13]	1996	The path of cerebral palsy can be partially explained by the rise in risk among extremely low birthweight and very preterm children whose survival is now improved, according to a study of recent papers, which is discussed in this article.
van Eyk et al. [14]	2018	Here, the difficulties in categorizing cerebral palsy and conditions similar to it are discussed.
McMichael et al. [15]	2015	Numerous genes have been linked to the genetic component of cerebral palsy causation, which has been hypothesized.
Rethlefsen et al. [16]	2010	There have been several classification schemes presented while discussing the categorization of cerebral palsy.
Bialik and Givon [17]	2009	There have been several classification schemes presented while discussing the categorization of cerebral palsy.
Dzienkowski et al. [18]	1996	The objective of this article is to provide advanced practice nurses with the necessary tools to provide patient and family care by examining the causes, pathophysiology, Swedish system diagnostic classification, clinical manifestations, and treatment approaches for cerebral palsy.
Krigger [19]	2006	This article discusses valid and trustworthy assessment techniques to establish baseline functions and track developmental progress that have led to a growing corpus of evidence-based recommendations for cerebral palsy.
Brandenburg et al. [20]	2019	This article focuses on the critical assessment of cerebral palsy and the use of animal models to comprehend the condition.
Paneth [21]	2008	This article discusses the symptoms of cerebral palsy and the different tests that must be done to get a diagnosis.
Chin et al. [22]	2020	This article discusses the fundamentals of medicine and surgery treatment for cerebral palsy.
Sankar and Mundkur [24]	2005	In order to properly organize and implement an early intervention program, a full neurodevelopmental assessment of the child with cerebral palsy should include an examination of any related deficiencies.
Dodge [25]	2008	According to the article, a pediatric healthcare provider's other responsibilities include assisting families in managing persistent health difficulties that may occur and instilling in them the belief that they are doing everything possible and necessary to ensure their kid achieves his or her full potential.
Graham et al. [26]	2019	The care of cerebral palsy frequently presents difficulties with stiffness and dystonia, pain control, hip monitoring, sleep, eating, swallowing, and nutrition.
Wilson et al. [27]	2022	The establishment of a cerebral palsy curriculum and exposure to cerebral palsy clinics may enhance training, translating to improved treatment for cerebral palsy sufferers, according to the article's point of view.
Aisen et al. [28]	2011	The article expresses its concern over the subject of therapies that cater to the requirements of adults aging with cerebral palsy.
Enslin et al. [29]	2019	The article talks about the neurophysiology of cerebral palsy and selective dorsal rhizotomy.
Park et al. [30]	1993	In order to treat spastic cerebral palsy, the article describes a kind of selective dorsal rhizotomy that involves sectioning the dorsal spinal roots immediately caudal to the conus medullaris.
Peacock and Staudt [31]	1991	The article tells us about selective posterior rhizotomy as a neurological procedure in the treatment of cerebral palsy.
Koy and Timmermann [32]	2017	Setting eligibility standards aids in strengthening the DBS management of youngsters.
Lumsden et al. [33]	2013	This study sought to determine the effects of age, contracture, and dystonia etiology on the outcome of deep brain stimulation (DBS) surgery.
Lin et al. [34]	2014	The article talks about the impact of dystonia in childhood.
Deli et al. [35]	2012	The article discusses bilateral pallidal deep brain stimulation (DBS), a well-researched therapeutic option for primary global and segmental dystonia.

## Conclusions

Cerebral palsy, despite being classified as a developmental brain injury, cannot be consistently or clearly linked to certain brain lesions. It is a complicated, diverse condition. The existence of a developmental disruption leading to motor or postural deficits is the only feature of cerebral palsy. Few animal models of cerebral palsy have successfully replicated the motor symptoms of the condition, an important aspect of the diagnosis, despite the fact that many have concentrated on inducing a brain lesion. It is believed that with the development of biomarker discovery, our comprehension of the etiopathophysiology of cerebral palsy will also grow, opening up additional potential for creating innovative prognoses and therapies. It is linked to pregnancy and birth problems, including "birth asphyxia" and low birth weight.
